# An Unusual Case of Large Cell Neuroendocrine Cancer of the Gallbladder With Mixed Adenocarcinoma Component in a Patient With Pancreatobiliary Maljunction

**DOI:** 10.7759/cureus.37398

**Published:** 2023-04-10

**Authors:** Anton Fick, Kayla Tran, Harsh Kandpal, Matthew Burge, Manju Chandrasegaram

**Affiliations:** 1 General Surgery, The Prince Charles Hospital, Brisbane, AUS; 2 Pathology, The Prince Charles Hospital, Brisbane, AUS; 3 Radiology, The Prince Charles Hospital, Brisbane, AUS; 4 Medical Oncology, The Prince Charles Hospital, Brisbane, AUS

**Keywords:** large cell neuroendocrine tumour, pancreatobiliary maljunction, gallbladder neuroendocrine carcinoma, gallbladder neuroendocrine tumour, gallbladder neuroendocrine neoplasm

## Abstract

Gallbladder neuroendocrine tumors (GB-NETs) and gallbladder neuroendocrine carcinomas (GB-NECs) are rare forms of neuroendocrine neoplasms (NENs). Most GB-NENs present as incidental findings or as gallbladder polyps in the course of investigation of nonspecific symptoms such as upper abdominal pain and nausea. Given the rarity of GB-NENs, only a few small case series are currently available describing this entity, and even fewer guiding consensus on standard treatment and the role of adjuvant therapy.

We present the case of a 65-year-old South American female referred for a chronic history of intermittent post-prandial epigastric pain, bloating, nausea, and occasional diarrhea. Pancreaticobiliary maljunction with dilation was present and she was diagnosed with primary gallbladder large cell neuroendocrine carcinoma (GB-LCNEC) mixed with a minor component of gallbladder adenocarcinoma.

## Introduction

Gallbladder cancers account for only 0.5% of all malignant cancers with an incidence of ~ 1.2 per 100,000 [1-3]. Neuroendocrine neoplasms (NENs) are a group of rare heterogeneous tumors representing ~ 2% of all malignancies and are functionally divided into two groups based on their clinical behavior, histology, and proliferation rate. Neuroendocrine tumors (NETs) include low to intermediate-grade well-differentiated tumors, and neuroendocrine carcinomas (NECs) include high-grade poorly differentiated tumors [4,5]. Neuroendocrine neoplasms most commonly affect the gastrointestinal system accounting for ~ 66% of all NENs [2,5,6]. Rarer still are primary gallbladder neuroendocrine neoplasms (GB-NENs) comprising only 0.5% to 2% to 3% of all gallbladder cancers [1,7-9] and 0.2% to 0.5% of all NENs [2,5,8,10]. The incidence of GB-NENs is reported to be less than 0.74 per 100,000 while gallbladder neuroendocrine carcinomas (GB-NECs) account for only 0.2% of all gastrointestinal NECs (GI-NECs), 0.5% of all NECs, and 2.1% of all gallbladder carcinomas [5,6].

Gallbladder neuroendocrine neoplasms are thought to arise from multipotent stem cells or neuroendocrine cells following gastric or intestinal metaplasia as a consequence of chronic inflammation secondary to gallstones [1,2,5,8,10-13]. Gallbladder neuroendocrine neoplasms were reported as mixed in four of 10 cases reported in a case series [6]. A retrospective review of the American National Cancer Database by Ayabe et al. in 2019, identified 754 patients with GB-NET. Of these, 480 (64%) underwent resection with most undergoing a simple cholecystectomy (n=431, 90%). A minority received multimodal therapy (n=145, 21%). Patients who were older and had large cell histology or positive margins had poorer survival following surgery [9].

## Case presentation

A 65-year-old female patient of South American descent was referred to our surgical outpatient department with a one-year history of intermittent post-prandial epigastric pain, nausea, bloating, and occasional diarrhea. She was a non-smoker and a non-drinker. She was otherwise fit and well apart from having previously undergone a cesarean section followed by a total abdominal hysterectomy. There was no family history of malignancy.

**Figure 1:**
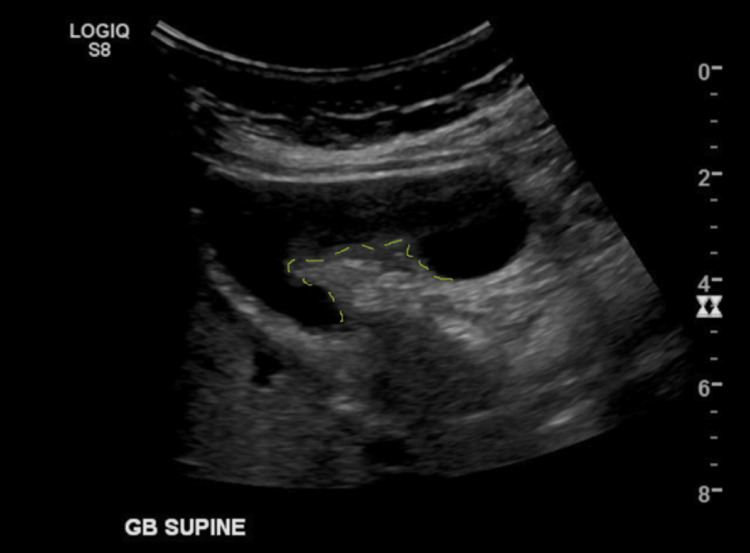
Unusual appearance of the gallbladder on ultrasound which was reported as possible adenomyomatosis

On examination, the patient's abdomen was soft and non-tender. Her blood tests revealed mildly deranged liver function tests with a bilirubin of 16 umol/L (reference range <20), alkaline phosphatase (ALP) 87 U/L (reference range 30-110), gamma-glutamyl transferase (GGT) 17 U/L (reference range <38), alanine transaminase (ALT) 48 U/L (reference range <34), and aspartate aminotransferase (AST) 38 U/L (reference range <31). An ultrasound performed by her general practitioner revealed an unusual gallbladder appearance and irregular thickening of her gallbladder wall up to 8 mm suspicious for a neoplastic process (Figure 1). An urgent magnetic resonance cholangiopancreatography (MRCP) and tumor markers were organized. She had a raised cancer antigen (CA) 19-9 at 51 kU/L (reference range <35).

**Figure 2:**
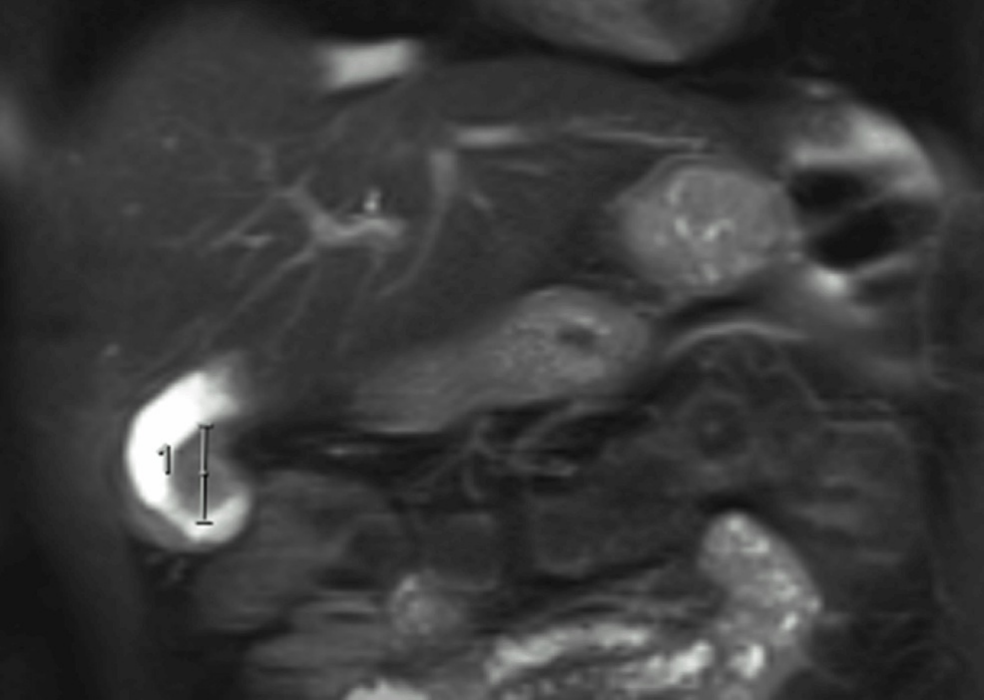
Magnetic resonance cholangiopancreatography (MRCP) demonstrating a mass in the gallbladder

**Figure 3:**
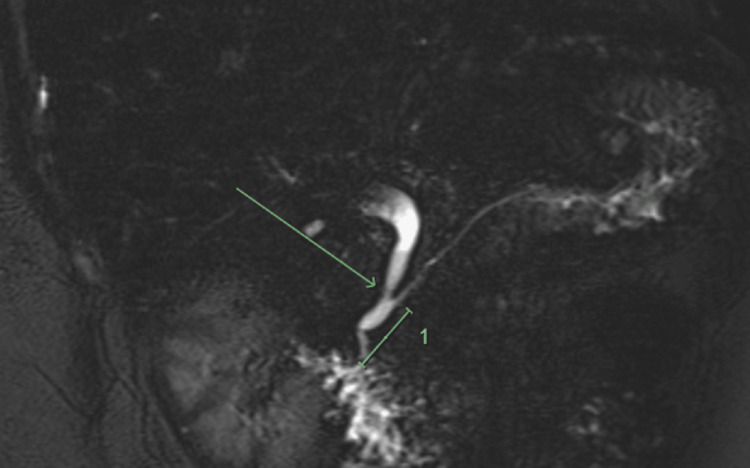
Magnetic resonance cholangiopancreatography (MRCP) demonstrating an anomalous pancreatobiliary junction with a long common channel

The MRCP revealed a 10 x 15 x 23 mm focal gallbladder wall thickening in the medial aspect of the fundus which was isointense on T1 with restricted diffusion suspicious for a neoplastic process (Figure 2). There were no internal T2 hyperintensities within this region to suggest cystic spaces or Rokitansky Aschoff sinuses. The MRCP also demonstrated an anomalous pancreatobiliary junction also known as pancreatobiliary maljunction (PBM) with a 23 mm long common channel (Figure 3). There was a focal narrowing of the distal common bile duct (CBD) at the site where it joined the dilated common channel (type C anomalous junction) and there was no evidence of cholelithiasis or choledocholithiasis.

**Figure 4:**
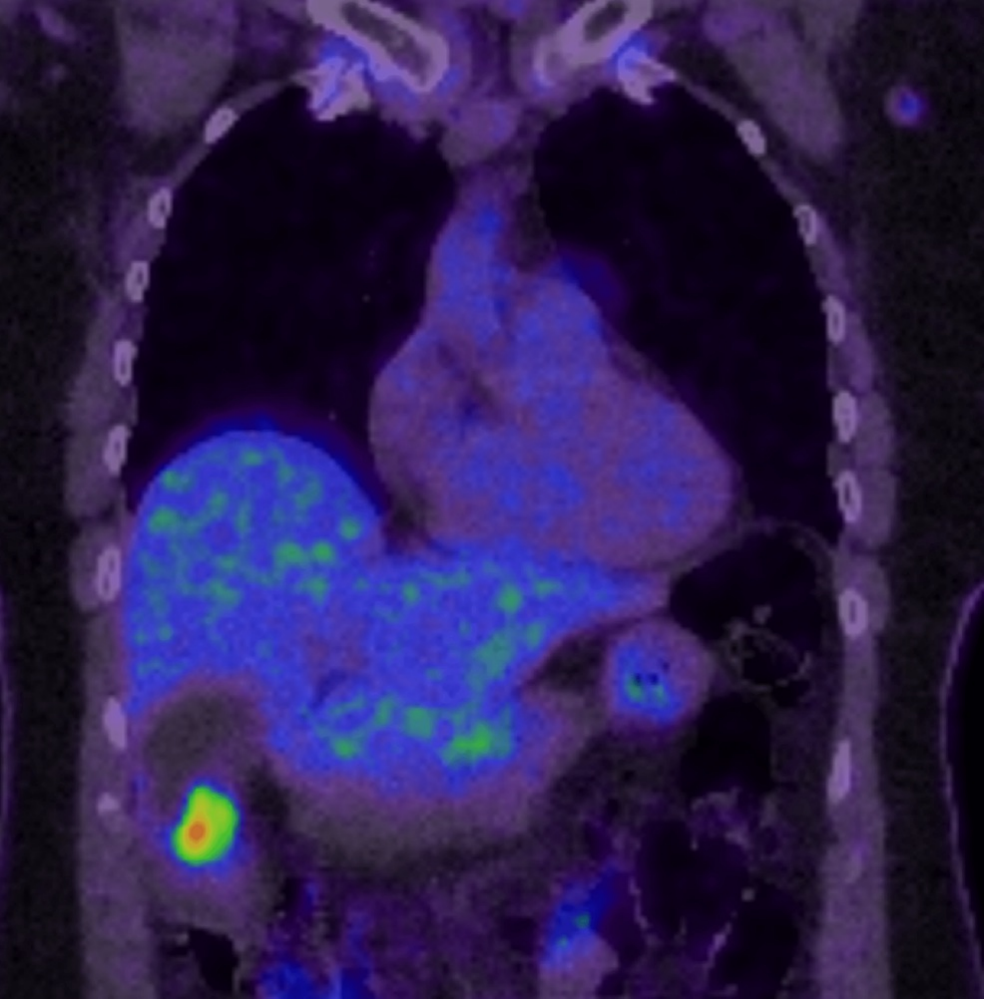
Moderate to intense fluorodeoxyglucose (FDG) avidity in the gallbladder body towards the fundus

**Figure 5:**
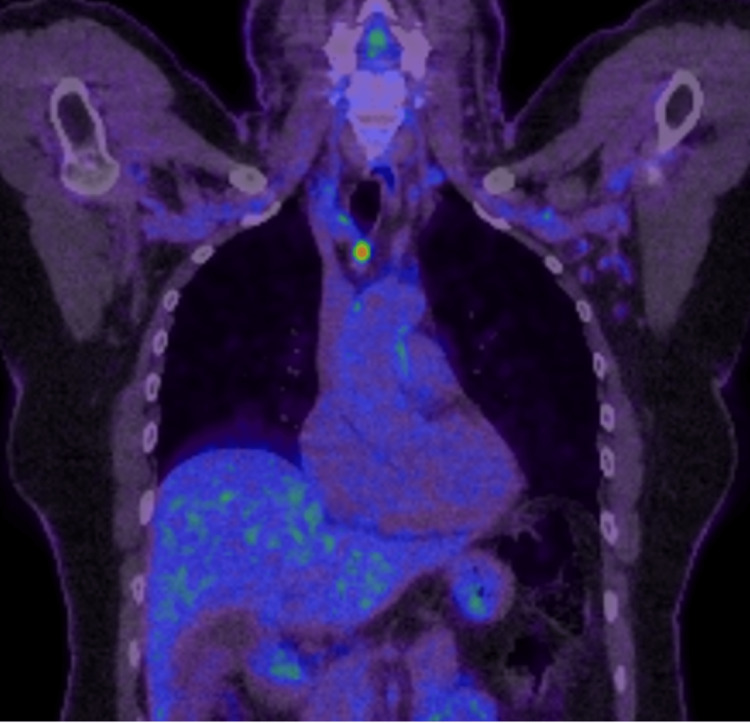
A single moderate to intensely fluorodeoxyglucose (FDG) avid upper mediastinal lymph node, immediately anterior to the trachea

A positron emission tomography (PET) was organized and demonstrated moderate to intense fluorodeoxyglucose (FDG) avidity in the area of abnormal enhancement and wall thickening in the gallbladder body (towards the fundus) as seen in Figure 4. There was also no evidence of FDG avid nodal metastases in the abdomen or peritoneal metastases. There was a single moderate to intensely FDG avid upper mediastinal lymph node, immediately anterior to the trachea (2R). This was suspicious of a pathologic node but distant nodal metastasis from the gallbladder was felt unlikely (Figure 5). There was also focal moderate FDG uptake in the distal thoracic esophagus.

The patient underwent a gastroscopy and biopsy of the lower esophagus to evaluate the PET area of avidity and this revealed Los Angeles (LA) classification grade B esophagitis. There was no dysplasia or malignancy present. Endoscopic bronchial ultrasound-guided biopsy of the PET avid pre-tracheal node was consistent with a reactive lymph node with no malignant cells identified.

**Figure 6:**
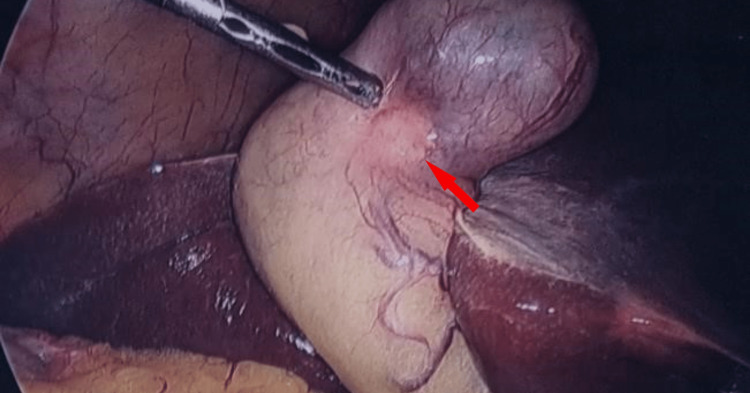
Intraoperative photo of the gallbladder with an arrow pointing at the tumor

**Figure 7:**
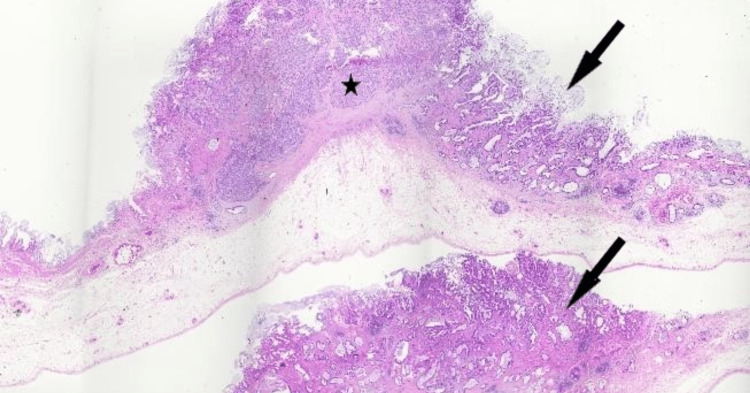
Tumour cross sections showing neuroendocrine carcinoma component (asterisk) and adenocarcinoma component (arrow); hematoxylin and eosin stain

**Figure 8:**
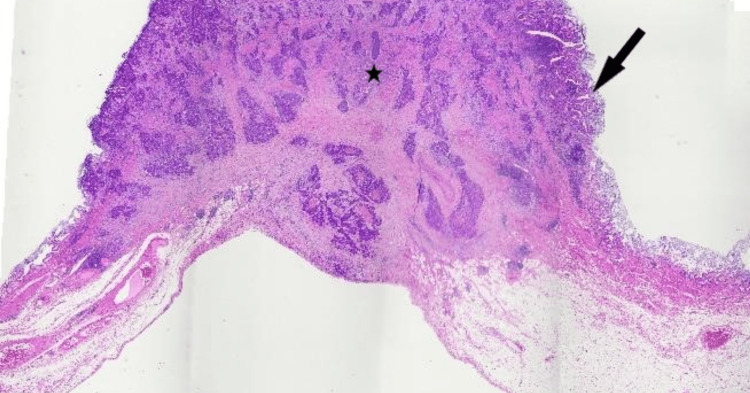
Tumour cross-section with mainly neuroendocrine carcinoma component (asterisk) and a small amount of adenocarcinoma (arrow); hematoxylin and eosin stain

**Figure 9:**
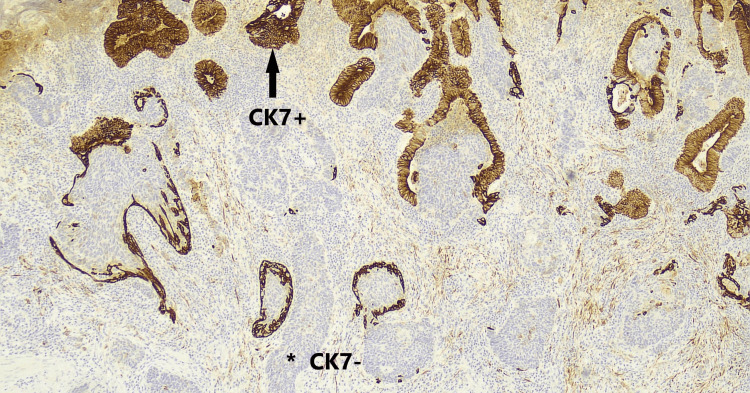
Cytokeratin 7 (CK7) immunohistochemical stain. Positive brown staining (CK7+) glandular elements (arrow) represent CK7+ adenocarcinoma cells. Non-staining (CK-) grey islands of cells (*) represent the neuroendocrine carcinoma cells.

**Figure 10:**
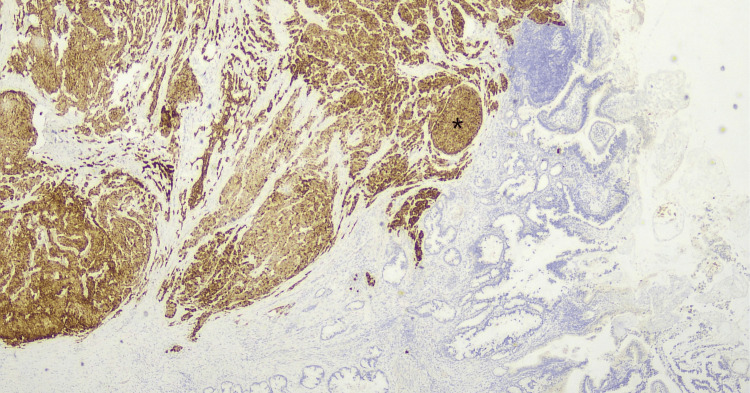
Chromogranin immunohistochemistry stain. The brown-stained tissue (asterisk) represents neuroendocrine carcinoma cells while the adenocarcinoma cells do not stain with chromogranin.

Given there was no evidence of distant or metastatic disease, the patient proceeded to have a radical cholecystectomy with a hand-assisted laparoscopic cholecystectomy, including a segment 4B/5 liver wedge resection and portal lymphadenectomy (Figure 6). Histology revealed a 32 mm pT2aN0 large cell neuroendocrine carcinoma of the gallbladder on the peritoneal aspect of the gallbladder with a mixed 20% component of adenocarcinoma (Figures 7, 8). The resected margins were clear and the hepatic aspect was not involved. Tumor clearance was reported to be 63 mm from the cystic duct margin and 13 mm from the hepatic parenchymal "radial" margin. The visceral peritoneum was not breached and the liver parenchyma was unremarkable. Although the lymphatic and perineural invasion was present, the cystic node was negative. The resected periportal tissue did not contain lymph nodes and in retrospect, we should have submitted it for re-evaluation but took the cystic node negativity to confirm node negative status. The resected periportal tissue did not contain portal lymph nodes and no malignancy was noted in the periportal tissue. Immunohistochemistry of the neuroendocrine carcinoma component demonstrated positivity for neuroendocrine markers cluster of differentiation (CD)56, synaptophysin, and chromogranin (Figures 9, 10). Immunohistochemistry did not demonstrate loss of staining for mismatch repair gene proteins. The Ki-67 proliferation index ranged from 20% to 40% in the large cell NEC component. 

**Figure 11:**
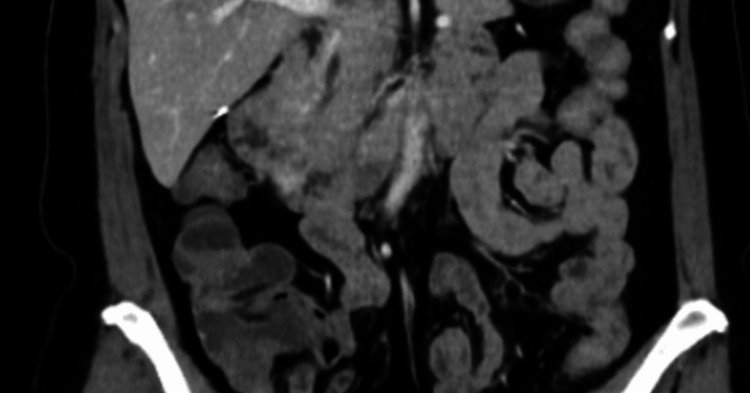
CT abdomen at the three-year follow-up showed no evidence of recurrence

The patient was referred to medical oncology and commenced on adjuvant capecitabine. This unfortunately led to an episode of capecitabine-induced coronary vasospasm two days into her treatment following which chemotherapy was ceased. She underwent three monthly reviews in the first year and then six monthly follow-up reviews with accompanying chest, abdomen, and pelvis progress CT scans at review appointments. She remains well and disease free three years postoperatively; her surveillance CT imaging demonstrated no evidence of loco-regional recurrence or malignancy (Figure 11) and her CA 19-9 is normal. 

## Discussion

Gallbladder cancers are rare and account for approximately 0.5% of all cancers [1]. Adenocarcinoma is the most common histological subtype accounting for between 90% to 98% of these cancers [1,7,11]. The literature suggests that NET of the gallbladder is rarer still representing 0.2% to 0.5% of all NETs and approximately 0.2% to 2% of all gallbladder cancers [7,8,10,14] and less than 1% of GB-NECs are functioning tumors [8].

The etiology and pathogenesis of GB-NEC are poorly understood although the literature suggests the malignant transformation of the gallbladder occurs through primarily two recognized sequences: the metaplasia-dysplasia-carcinoma sequence and the adenoma-carcinoma sequence although the former is favored [2,8,15-21]. Gallbladder cancer associated with cholelithiasis occurs predominantly via the metaplasia-dysplasia-carcinoma sequence. Metaplasia occurs in an attempt to defend against gallstone or other related tissue damage and chronic inflammation. Gallbladder epithelial metaplasia is common in relation to cholelithiasis and chronic cholecystitis and typically involves differentiation into pseudopyloric epithelium (59.5% to 95%) and intestinal epithelium (9.5% to 58.1%) [20]. Gallbladder metaplasia is identified in 66% of gallbladders with invasive carcinoma [20]. 

The WHO classification of NETs of the gastrointestinal tract and hepatopancreatobiliary system (Table 1) categorizes GB-NENs based on cellular differentiation guided by the mitotic rate (mitoses/2mm^2^) [22]. Eltawil et al. in 2010 described gallbladder-large cell NECs (GB-LCNECs) as “exceedingly rare” with only ~ 10 cases being described, and classified by the WHO as aggressive high-grade GB-NENs [2,22]. 

**Table 1:** The World Health Organisation classification of neuroendocrine tumors of the gastrointestinal and hepatopancreatobiliary systems NET: Neuroendocrine tumor, NEC: Neuroendocrine carcinoma, SCNEC: Small cell neuroendocrine carcinoma, LCNEC: Large cell neuroendocrine carcinoma

Terminology	Differentiation	Grade	Mitotic rate (mitoses/2 mm^2^)	Ki-67 Index
NET, grade 1	Well-differentiated	Low	<2	<3 %
NET, grade 2		Intermediate	2-20	3%-20 %
NET, grade 3		High	>20	>20 %
NET, small cell type (SCNEC)	Poorly differentiated	High	>20	>20 %
NEC, large cell type (LCNEC)		High	>20	>20 %
MiNEN	Well or poorly differentiated	Variable	Variable	Variable

As there are no neuroendocrine cells within normal gallbladder histology, the origin of gallbladder NET and mixed neuroendocrine-non-endocrine neoplasms (MiNEN) remains unclear although several theories currently exist. A widely accepted hypothesis is that both adenocarcinomas and neuroendocrine carcinomas derive from a common single biliary tree multipotent stem or progenitor cell subserving biliary tree regeneration [1,7,8,10,11].

Another hypothesis suggests gallbladder carcinomas and MiNEN may arise consequential to the classical adenoma-carcinoma sequence [1,15]. It is well documented that gastric and intestinal metaplasia changes with neuroendocrine cells occur secondary to chronic cholecystitis [1,10,13]. The literature also suggests the possibility of transformation of gallbladder adenocarcinoma to GB-NEC [21,23].

A third mechanism underlying biliary carcinogenesis in patients with PBM has been suggested to involve a chemically induced hyperplasia-dysplasia-carcinoma sequence secondary to reflux and stasis of bile mixed with pancreatic secretions in the bile duct and gallbladder with consequent gene mutations, particularly in K-ras and p53 [8,15,16,18,24,25]. Kanthan et al. suggest that “...almost all gallbladder NETs reportedly demonstrated co-existing cholelithiasis and chronic cholelithiasis and that less than 1% presented with functioning lesions such as carcinoid syndrome...” [8]. Gallbladder cancers associated with PBM are, however, reported to have lower incidences of associated cholelithiasis [3,15,19].

While many risk factors for gallbladder cancer are reported, it is well-recognized that South American ethnicity, cholelithiasis, and chronic cholecystitis are significant risk factors for gallbladder cancers [8]. The patient in our study is a South American but had no history of cholecystitis and no evidence of cholelithiasis. However, preoperative imaging demonstrated PBM with a type C anomalous junction and a 23 mm long dilated common channel. Although rarer, PBM is also a recognized risk factor for gallbladder cancers [8].

Pancreatobiliary maljunction represents a congenital anomaly of the pancreatobiliary tree resulting in a premature confluence of the bile and main pancreatic ducts outside the duodenal wall tending to form a long channel and is morphologically divided into PBM with or without biliary dilation [15,20,24-26]. The incidence of PBM in western populations is reported as 1:100,000 to 150,000 although the incidence is higher in Asia at 1 in 1000 [16,24]. This is in keeping with the lower combined incidence of bile duct and gallbladder cancer in Europe which is reported as 1.8 to 7 per 100,000.

A 2013 nationwide survey in Japan by Morine et al. involving a retrospective review of 2529 patients over 18 years, identified PBM in 1511 adult patients and 1018 pediatric patients [17]. Of the 1018 pediatric cases, 950 had PBM with biliary dilation and 68 had PBM without biliary dilatation. Biliary cancer was identified in one child (0.1%) with biliary dilation and was located in the bile duct. No biliary cancers occurred in those children without biliary dilation. Of the 1511 adults, 997 had biliary dilation and 514 had PBM without biliary dilation. In the adult cohort with PBM, biliary tree/gallbladder cancer occurred in 215/997 (21.6%) patients with biliary dilation, and 218/514 (42.4%) patients without biliary dilation. Of these cancers, the majority were gallbladder cancers which occurred in 134/215 (62.3%) patients with biliary dilation and 192/218 (88.1%) patients without biliary dilation. Combined gallbladder and bile duct cancers occurred in 10/215 (4.7%) patients with biliary dilation and 9/218 (4.1%) patients without biliary dilation [17].

A retrospective review of 142 gallbladders by Muraki et al. compared 76 gallbladders from patients with preoperative radiologically confirmed PBM to 66 gallbladders in patients without PBM on the preoperative radiologic investigation. Gallbladder carcinoma was identified in 22/76 (28.9%) of the patients with PBM. In contrast, no gallbladder cancers were identified in the gallbladders of patients without PBM [19].

A recent case series of 145 PBM patients conducted by Kamisawa (&) Honda in 2019, identified 73 patients with biliary dilation and 72 without biliary dilation. Bile duct and gallbladder cancer occurred in eight (11%) and 15 (21%) of the 73 patients with congenital biliary dilation, and in two (3%) and 49 (68%) of 72 patients without biliary dilation. Overall, 74 (51%) of patients with PBM in this series developed biliary tract cancer. Of those 74 patients, 64 (86.5%) developed gallbladder cancer [15]. These findings appear to correlate with the 2013 nationwide Japanese survey.

Pancreaticobiliary maljunction is reported to confer a 200-fold increased risk of gallbladder and bile duct cancers as well as an approximately 49-fold risk of pancreatic cancer. Given the high risk of malignant transformation in PBM, the Japanese Clinical Practice Guidelines for PBM recommends prophylactic cholecystectomy once PBM is diagnosed [15,19]. Morine et al. reported clinical features that were identified in patients with PBM included abdominal pain, back pain, jaundice, fever, vomiting, pale stool, as well as acute and chronic pancreatitis, cholangitis, liver dysfunction, biliary tree calculi, and raised amylase levels [3]. Diagnosis of PBM, however, requires endoscopic retrograde cholangiopancreatography (ERCP) or MRCP [15,19,24].

The relative risk for gallbladder carcinoma in patients with PBM is reported to be 167.2 and 419.6 times higher than in the general population [20]. Espinoza et al. also suggest gallbladder carcinogenesis in patients with PBM progresses through the metaplasia-dysplasia-carcinoma sequence secondary to chronic inflammation consequent to the stasis of mixed bile and refluxed pancreatic secretions [20]. The literature, however, also describes reflux-associated cholecystopathy to progress through a hyperplasia-dysplasia-carcinoma sequence in patients with PBM [15,18,19]. Muraki et al. describe this as a local chemical-induced hyperplasia-carcinogenesis pathway although intestinal and pyloric gland metaplasia was also reported in their case series. Intestinal metaplasia and pyloric metaplasia were identified in 24% and 70%, respectively, of all 76 gallbladders from patients with PBM compared to 6% and 48% of 66 gallbladders from patients without PBM, and with higher intestinal metaplasia rates in patients with PBM being statistically significant [19]. 

Gallbladder neuroendocrine carcinomas can be difficult to diagnose as there are no specific signs or symptoms. Niu et al. report early clinical features for GB-NET/C that mimic other GB cancers and include vague constitutional symptoms such as abdominal distension, pain, nausea, and other non-specific symptoms making diagnosis difficult [21]. Gallbladder neuroendocrine tumors/cancers with carcinoid syndrome may present with additional and more specific symptoms such as spasmodic abdominal pain, flushes, wheezing, diarrhea, and symptoms of right heart valve disease but it is recognized that such cases are particularly rare accounting for only 1% of all neuroendocrine carcinomas [21,23,27,28].

Tumour markers such as CA 19-9, CA 125, and carcinoembryonic antigen (CEA) are usually in the normal ranges. Ultrasound, CT, or MRI may aid in diagnosis but fail to distinguish between NEC and other forms of gallbladder cancer [6,21,23,27-29]. Definitive diagnosis can only be made based on tissue pathology and immunohistochemistry [6,21,23,28,29]. Treatment for suspected GB-NEC depends on the tumor stage and the literature suggests simple cholecystectomy is adequate in early in-situ disease. In contrast, late-stage disease in patients with tumor-nodes-metastasis (TNM) grade II, III, and IV tumors require radical cholecystectomy including cholecystectomy, wedge resection of the liver, and lymph node dissection [6,21,23,27-29].

Given the incidence of GB-NEC is low, few studies are available that report their chemotherapy regimen (Table 2). While there are no randomized prospective studies, Niu et al. and Shimono et al.suggest that while surgery is the best option, chemotherapy may prolong progression-free survival [27,30-32]. Chemotherapy response rates for rapidly growing tumors are reported to range from 20% to 60% [21]. The literature also suggests the use of chemotherapy as an alternative in patients who are medically unfit for surgery [21,27,30,31,33]. Platinum-based postoperative chemotherapy is used in several studies [5,28-31].

**Table 2:** Summary of case series survival rates in patients with GB-NEC that received surgery (with curative intent or palliative) and adjuvant chemotherapy GB-NEC: Gallbladder neuroendocrine carcinoma

Author	Operation description	Chemotherapy regime	Survival (days)	Overall median survival (days)
Chen et al., 2015 [6] (n=10)	Palliative subtotal cholecystectomy	Etoposide + cisplatin (8 cycles)	380	98.5
Liu et al., 2019 [29] (n=8)	Radical dissection of GB + Quadrate Lobe	Etoposide + cisplatin (4 cycles) + gemcitabine (2 cycles)	25 months - no recurrence	
	Radical dissection of GB	Irinotecan + cisplatin (1 cycle)	12 months - recurrence	
Chu et al., 2020 [30] (n=7)	Laparoscopic radical cholecystectomy	Etoposide + cisplatin (2 cycles)	504	138
	Laparoscopic radical cholecystectomy	Etoposide + carboplatin (4 cycles)	138	
	Open radical cholecystectomy	Etoposide + cisplatin (7 cycles)	267	
Yan et al., 2020 [31] (n=15)	Radical resection	Adjuvant etoposide + cisplatin		612
	Radical resection	Adjuvant etoposide + cisplatin		
	Radical resection	Adjuvant etoposide + cisplatin		
	Radical resection	Adjuvant etoposide + cisplatin		
	Radical resection	Post-recurrence etoposide + cisplatin		
	Radical resection	Post-recurrence etoposide + cisplatin		
	Radical resection	Post-recurrence etoposide + cisplatin		
Do et al., 2021 [33] (n=21)	Extended cholecystectomy + right hepatectomy	Etoposide + cisplatin	496	166
	Radical cholecystectomy	Etoposide + cisplatin	416	
	Radical cholecystectomy	Etoposide + cisplatin	649	
	Extended cholecystectomy + bisegmentectomy	Etoposide + cisplatin	222	
	Laparoscopic cholecystectomy	Etoposide + cisplatin (palliative)	105	
	Extended cholecystectomy + segmentectomy	Etoposide + cisplatin	460	
	Radical cholecystectomy	Etoposide + cisplatin	237	

Niu et al. described statistically significant higher progression-free survival rates in patients with GB-NECs treated with octreotide compared to a placebo group. Molecular targeted therapy with sunitinib is also gathering interest as it has demonstrated a statistically significant extension of progression-free survival in pancreatic-NETs by interfering with vascular endothelial growth factor (VEGF)-mediated neovascularization [21]. Niu et al. also describe the use of radioactive isotopes such as Y90 and Lu177 that provide local inhibition of tumor growth [21].

## Conclusions

Our patient is a Colombian female with PBM (with biliary dilation), no history or evidence of cholelithiasis or cholecystitis, and was found to have large cell GB-NEC. Incidence of both large cell GB-NECs and PBM without biliary dilation are low making this case a rare and interesting gallbladder malignancy. Our patient has remained disease-free for 21 months following radical resection of this rare cancer without adjuvant therapy. The literature is evolving to guide the role of adjuvant chemotherapy which appears to favor a platinum-based regimen.
